# Polyphenol Composition and (Bio)Activity of *Berberis* Species and Wild Strawberry from the Argentinean Patagonia

**DOI:** 10.3390/molecules24183331

**Published:** 2019-09-12

**Authors:** Melina F. Chamorro, Gabriela Reiner, Cristina Theoduloz, Ana Ladio, Guillermo Schmeda-Hirschmann, Sergio Gómez-Alonso, Felipe Jiménez-Aspee

**Affiliations:** 1 Laboratorio Ecotono, INIBIOMA (CONICET-Universidad Nacional del Comahue), Bariloche 8400, Río Negro, Argentina; 2 Laboratorio de Cultivo Celular, Facultad de Ciencias de la Salud, Universidad de Talca, Talca 3460000, Region del Maule, Chile; 3 Laboratorio de Química de Productos Naturales, Instituto de Química de Recursos Naturales, Universidad de Talca, Talca 3460000, Region del Maule, Chile; 4 Instituto Regional de Investigación Científica Aplicada, Departamento de Química Analítica y Tecnología de Alimentos, Universidad Castilla-La Mancha, 13005 Ciudad Real, Spain; 5 Departamento de Ciencias Básicas Biomédicas, Facultad de Ciencias de la Salud, Universidad de Talca, Talca 346000, Región del Maule, Chile

**Keywords:** *Berberis microphylla*, *Berberis darwinii*, *Fragaria chiloensis* ssp. *chiloensis* f. patagonica, HPLC-DAD-ESI-MS^n^, anthocyanins, phenolic compounds, antioxidant capacity, Patagonia

## Abstract

The Argentinean Patagonia berries *Berberis microphylla*, *Berberis darwinii*, and *Fragaria chiloensis* ssp. *chiloensis* f. patagonica were investigated for their polyphenol content and composition by means of liquid chromatography coupled to diode array detection and electrospray ionization tandem mass spectrometry. The in vitro antioxidant activity and inhibition of metabolic syndrome-associated enzymes (α-glucosidase, α-amylase, and lipase) of the fruit extracts was assessed. The most complex polyphenol profile was found in the *Berberis* samples, with 10 anthocyanins, 27 hydroxycinnamic acids, 3 proanthocyanidins, 2 flavan-3-ol, and 22 flavonols. *Fragaria* presented four anthocyanins, nine ellagitannins, two proanthocyanidin dimers, one flavan-3-ol, and five flavonols. The *Berberis* samples showed the best antioxidant capacity, while *Fragaria* displayed better activity against α-glucosidase and lipase. The phenolic content and composition of the Argentinean Patagonia berries was similar to that reported for Chilean samples but with some chemical differences between Eastern (Argentina) and Western (Chile) Patagonia. The data obtained supports the consumption of these berries as sources of beneficial polyphenols.

## 1. Introduction

**Figure 1:**
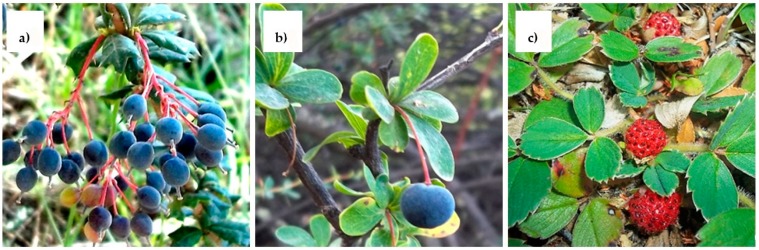
Fruits from Argentinean Patagonia berries: (**a**) *Berberis darwinii*, (**b**) *Berberis microphylla*, and (**c**) *Fragaria chiloensis*.

Edible wild plants play a relevant role in rural and indigenous communities around the world and have been used as food and medicine since ancient times [1]. Among them, wild berries are recognized worldwide as healthy foods, contributing to the prevention of several diseases [2]. Their beneficial health properties are linked to their antioxidant properties and their ability to protect from the oxidative effects of free radicals [3]. The use of wild berries in the Argentinean and Chilean Patagonia has been recently reviewed [4]. Among them, the “calafates or michays” *Berberis microphylla* G. Forst (Berberidaceae), *Berberis darwinii* Hook. (Berberidaceae), and the wild strawberry *Fragaria chiloensis* ssp. *chiloensis* f. patagonica (L.) Mill. (Rosaceae) are relevant for their widespread use (Figure 1) [5]. Most of the Patagonian territory is located from the Eastern Andean slopes to the Atlantic Ocean in Argentina. However, little is known on the chemical profiles of Argentinean Patagonia berries, in spite of them being integrated into the culture and traditional cuisine of Southern Argentina [4]. The fruits of *B. microphylla* sampled in Chile show a high content of polyphenols and a high antioxidant capacity [6]. At present, the Patagonian native berries are still underutilized fruit species and their development into new crops has been pointed out as relevant for the diversification of agricultural production of Argentina [7]. The fruits of calafate and native strawberry can be consumed fresh or processed into preserves, jams, and ice-creams, as well as to prepare alcoholic and non-alcoholic beverages. *Berberis* is a relevant genus for several cultures because of its medicinal and food value [8]. It includes almost 500 species distributed worldwide [8,9]. In the last decades, the studies on chemical composition of its species have been focused on berberine and other alkaloids, mostly reported in roots and bark [9,10]. There is an increasing interest in *Berberis* fruits, as they are a rich source of phenolic compounds [11,12,13]. The main phenolic compounds in fruits of Chilean *Berberis* species are anthocyanins, mainly delphinidin, cyanidin, petunidin, peonidin, and malvidin glycosides [14]. *Berberis* fruits showed higher antioxidant capacity than other Chilean wild berries [15]. The strong antioxidant capacity has been associated with their high anthocyanin content [14]. The fruits from *Fragaria chiloensis* ssp. *chiloensis* f. patagonica are consumed in Argentinean Patagonia and have been used in human and veterinary medicine [16]. The antioxidant activity of Chilean *F. chiloensis* samples has been related to the anthocyanin and ellagitannin content of the fruits [17].

The (bio)activity and chemical profiles of Patagonian wild berries have been carried out mainly for Chilean collections [18,19,20,21,22]. Differences in the composition and bioactivity of Patagonian berries occurring in both sides of the Andes Mountains have been described [18,23], and additional differences should be expected. The aim of the present work was to describe the polyphenol content and composition, antioxidant, and inhibitory activity towards metabolic syndrome-associated enzymes of different populations from Argentinean Patagonia berries (“calafates” and wild strawberry), and to compare the results with those reported for the Chilean samples.

## 2. Results and Discussion

Five samples from *Berberis microphylla*, two from *B. darwinii*, and two from *Fragaria chiloensis* were studied. Figure 1 shows the wild fruits from the selected species. Amberlite XAD-7^®^ is a non-ionic macroreticular resin that adsorbs and releases ionic species through hydrophobic and polar interactions. The clean-up steps allow for the removal of sugars, salts, and small organic acids, and thus enriches the extract in polyphenols. The moisture and yield of extraction are shown in Table 1. The extraction yields obtained are similar to those reported by Ruiz et al. [21]. These authors reported that the extraction of polyphenolic compounds was optimal when 93% methanol (MeOH) in acidified water was used as the solvent. Interestingly, the sonication time and washing steps showed no significant influence in the extraction yield.

### 2.1. Characterization of Individual Components by High Performance Liquid Chromatography Coupled to Diode Array Detector and Electrospray Ionization Mass Spectrometry (HPLC-DAD-ESI-MS^n^)

Patagonian berries are an important source of phytochemicals with potential health-promoting properties [4], in particular the genus *Berberis*. The phytochemistry and pharmacological properties of several *Berberis* species was reviewed by Srivastava et al. [9] and Mokhber-Dezfuli et al. [10]. In the same way, the chemical composition and health benefits of *Fragaria chiloensis* have been recently reviewed [4]. However, little is known on the chemical profiles of *Berberis* and *Fragaria* species growing in the Argentinean Patagonia. The phenolics occurring in the different samples were identified by the UV spectra, retention time, and mass spectrometry by HPLC-DAD-ESI-MS^n^ analysis. The results are presented below.

#### 2.1.1. Anthocyanins

**Figure 2:**
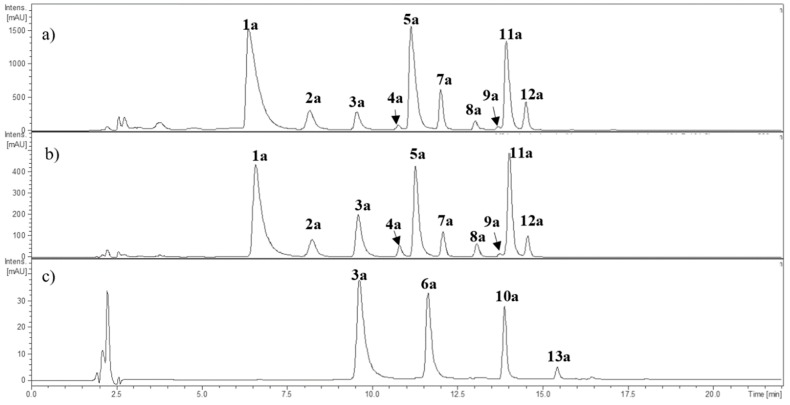
HPLC-DAD chromatogram (520 nm) of the anthocyanins from (**a**) *Berberis microphylla*, (**b**) *Berberis darwinii*, and (**c**) *Fragaria chiloensis*. The numbers correspond to Table 2.

The anthocyanin obtained by solid phase extraction (SPE) fractionation was analyzed at 500–520 nm and the MS^n^ fragmentation pattern in the positive ion mode [M + H]^+^. The SPE cartridge PCX^®^ (Agilent Bond Elut Plexa, Agilent, Santa Clara, CA, USA) combines cationic exchange with reverse-phase adsorption of polyphenolic compounds in order to retain anthocyanins and recover non-charged polyphenols. Anthocyanins can then be recovered from the cartridge for further analyses. The retention time of the compounds was used for matching and comparison when commercial standards were available. The chromatographic anthocyanin profiles of *Berberis microphylla*, *B. darwinii*, and *Fragaria chiloensis* are shown in Figure 2. The HPLC-DAD-ESI-MS^+^ data on anthocyanins is summarized in Table 2. Most of the anthocyanins detected showed the neutral loss of 162 amu, supporting the occurrence of a hexoside, or a neutral loss of 308 amu, in agreement with rutinoside. Compounds **1a** and **2a** showed a common fragment ion (MS^2^) at *m*/*z* 302.9 amu in agreement with delphinidin. Compounds **3a** and **4a** showed a MS^2^ fragment ion at *m*/*z* 286.9 amu, and compounds **5a** and **7a** a common MS^2^ fragment ion at *m*/*z* 316.9 amu, in agreement with cyanidin and petunidin, respectively. Compounds **8a** and **9a** showed the presence of a MS^2^ fragment ion at *m*/*z* 300.9 amu, and compounds **11a** and **12a** presented a common MS^2^ fragment ion at *m*/*z* 331.03 amu, characteristic of peonidin and malvidin, respectively. All the listed anthocyanins were detected in *B. microphylla* and *B. darwinii* (Table 2).

Ruiz et al. [14] described the anthocyanin profile of Chilean *B. microphylla*, reporting 18 anthocyanins, including delphinidin, cyanidin, petunidin, peonidin, and malvidin derivatives. The sugar moieties were glucose, rutinose, and dihexose.Ramirez et al. [15] described the occurrence of delphinidin, petunidin, peonidin, and malvidin anthocyanins in *B. microphylla* samples collected in the Región de Ñuble (central southern Chile), but not the presence of cyanidin. Ruiz et al. [24] reported the isolation and characterization of 3,7-β-*O*-diglucosides of delphinidin, petunidin, and malvidin, and suggested the occurrence of the same derivatives of cyanidin and peonidin. The dihexosides, however, were not detected in our Argentinean samples, and are so far a difference between the eastern and western Patagonia population of the species. The *F. chiloensis* phenolic enriched extracts (PEEs) showed the presence of cyanidin hexoside (**3a**) and the additional signal of compound **6a** with a [M − H]^−^ molecular ion at *m*/*z* 433.3 amu, with MS^2^ fragment ion at *m*/*z* 271.2 amu and λ_max_ at 502 nm, characteristic of pelargonidin [25]. The compound was identified as pelargonidin hexoside. Two other anthocyanins, compounds **10a** and **13a**, were detected in this species. These compounds showed the neutral loss of 248 amu, leading to a MS^2^ fragment ion at *m*/*z* 286.9 and 271.1 amu, respectively, and were tentatively identified as malonyl hexosides of cyanidin and pelargonidin, respectively. In *F. chiloensis* ssp. *chiloensis* f. patagonica, Simirgiotis et al. [26] described the same four main anthocyanins, namely: cyanidin-3-*O*-glucoside, pelargonidin-3-*O*-glucoside, cyanidin-malonyl-glucoside, and pelargonidin-malonyl-glucoside. Overall, the profile of anthocyanins in Argentinean samples is similar to that found in the Chilean (western Patagonia) collections.

#### 2.1.2. Hydroxycinnamic Acids (HCAs)

**Figure 3:**
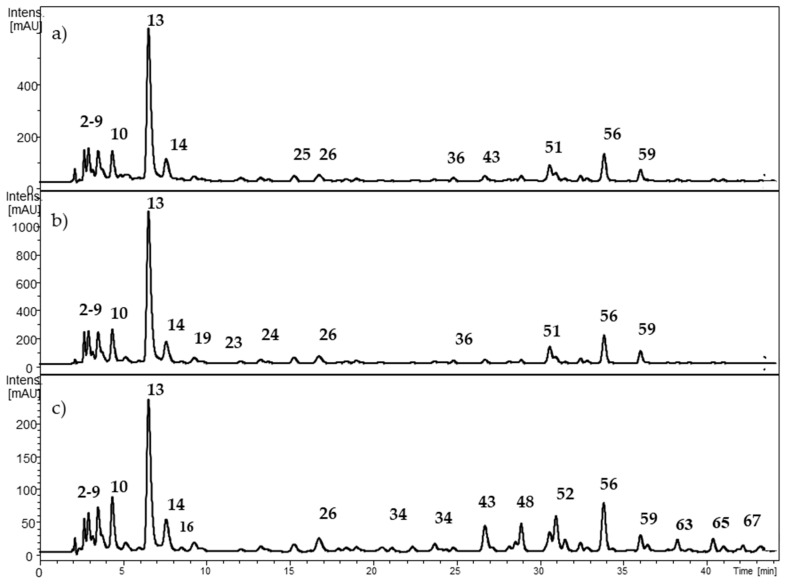
HPLC-DAD chromatogram from *Berberis microphylla* at (**a**) 280 nm; (**b**) 320 nm; and (**c**) 360 nm. The numbers correspond to Table 3.

**Figure 4:**
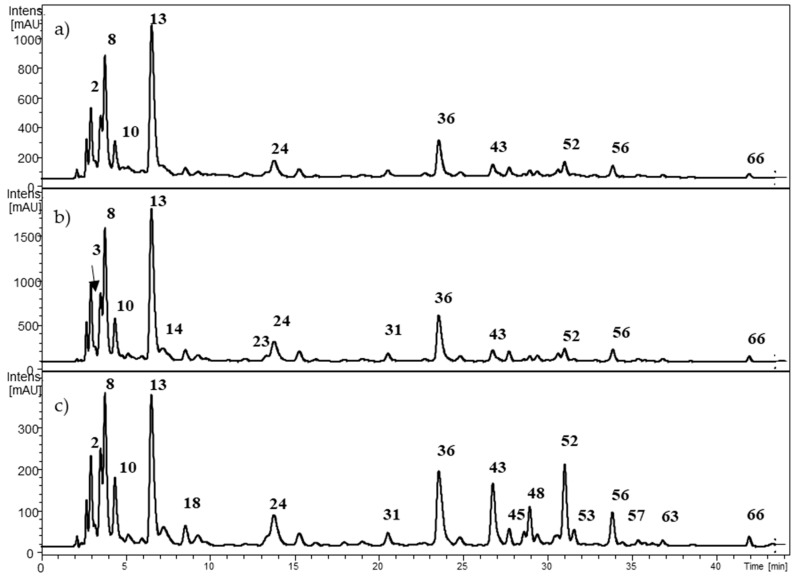
HPLC-DAD chromatogram from *Berberis darwinii* at (**a**) 280 nm; (**b**) 320 nm; and (**c**) 360 nm. The numbers correspond to Table 3.

**Figure 5:**
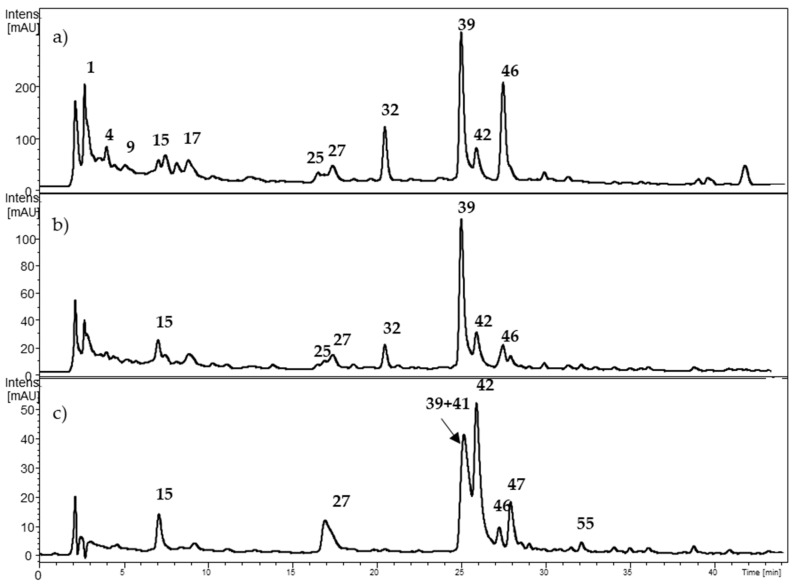
HPLC-DAD chromatogram from *Fragaria chiloensis* at (**a**) 280 nm; (**b**) 320 nm; and (**c**) 360 nm. The numbers correspond to Table 3.

The profile of non-anthocyanin polyphenols is depicted in Figure 3 for *Berberis microphylla*, Figure 4 for *B. darwinii*, and Figure 5 for *Fragaria chiloensis*. The HCA identity assignment was carried out in the non-anthocyanin polyphenol extract by the analysis of the UV-VIS profile, MS^n^ fragmentation pattern in the negative ion mode [M − H]^−^, and the hierarchical scheme proposed by Clifford et al. [27]. The retention time was used for matching when commercial standards were available. Table 3 shows the retention time and spectral data of HCAs found in the samples. A total of 27 HCAs with λ_max_ at 320 nm were detected in the Argentinean Patagonia samples. Most of them were found in both *Berberis* species. HCAs were not detected in our samples of *F. chiloensis* ssp. *chiloensis* f. patagonica. In the native Chilean white strawberry (*F. chiloensis* ssp. *chiloensis* f. chiloensis), Cheel et al. [28] isolated three cinnamoyl hexosides. The presence of caffeoyl, coumaroyl, and feruloyl hexosides have been reported in commercial strawberries [29]. More samples of *F. chiloensis* from the Argentinean Patagonia are needed to confirm this interesting difference.

Compounds **2** and **5** showed a common [M − H]^−^ molecular ion at *m*/*z* 370.9 amu, with MS^2^ fragment ions at *m*/*z* 209 and 191 amu, indicating the presence of a hexaric acid core (glucaric, mannaric, or galactaric acids) [30]. The neutral loss of 162 amu agreed with a caffeoyl moiety. Thus, the compounds **2** and **5** were assigned as caffeoyl hexaric acid isomers 1 and 2, respectively. The compounds **14**, **19**, **24**, **29**, **33**, **36**, **44**, and **49** showed a common [M − H]^−^ molecular ion at *m*/*z* 533.0 amu. Two consecutive losses of 162 amu indicated the presence of two caffeoyl moieties, leading to a MS^2^ fragment ions at *m*/*z* 209 and 191 amu. The compounds were identified as dicaffeoyl hexaric acid isomers. Compounds **3** and **8** showed a common [M − H]^−^ molecular ion at *m*/*z* 352.9 amu, leading to a MS^2^ fragment ion at 190.6. Following the hierarchical scheme proposed by Clifford et al. [27] the compounds were identified as 5- and 3-caffeoylquinic acids, respectively. The identity of both compounds was confirmed with commercial standards. In addition, compounds **10**, **13**, **16**, and **23** showed a similar fragmentation pattern and were tentatively identified as caffeoylquinic acid isomers. Compounds **26**, **37**, **51**, and **59** showed a common [M − H]^−^ molecular ion at *m*/*z* 514.9 amu, followed by two consecutive losses of 162 amu, leading to the MS^2^ fragment ion at *m*/*z* 190.68 amu. The compounds were tentatively identified as dicaffeoylquinic acid isomers [27]. Compounds **11** and **22** presented a [M − H]^−^ molecular ion at *m*/*z* 337.2 amu, leading to a MS^2^ fragment ion at 190.6 amu. The compounds were identified as 3-*p*-coumaroylquinic acid (**11**) and 5-*p*-coumaroylquinic acid (**22**), respectively [27]. Compounds **18**, **21**, **28**, **35**, and **38** presented a common [M − H]^−^ molecular ion at *m*/*z* 367.3 amu. The neutral loss of a feruloyl moiety to the MS^2^ fragment ion at 191 amu allowed the tentative identification of both compounds as feruloylquinic acid isomers.

Ruiz et al. [21] described the presence of 20 different HCAs in ripe fruits of *Berberis microphylla* collected in the Chilean Patagonia. The identity of the compounds was determined by HPLC-MS, including: four caffeoylglucaric acid isomers, seven caffeoylquinic acid isomers, two dicaffeoylglucaric acid isomers, one coumaroylquinic acid, two feruloylquinic acid isomers, three dicaffeoylquinic isomers, and one feruloylcaffeoylquinic acid.

#### 2.1.3. Ellagitannins

Ellagitannins were only detected in the non-anthocyanin fraction of *F. chiloensis* ssp. *chiloensis* f. patagonica (Table 3). The ellagic acid derivatives were identified by the common [M − H]^−^ molecular ion at *m*/*z* 300.5 amu. Compound **27** showed the neutral loss of 162 amu, in agreement with ellagic acid hexoside. Compound **39** showed a [M − H]^−^ molecular ion at *m*/*z* 435.0 amu followed by the neutral loss of 132 amu, in agreement with ellagic acid pentoside. Compound **46** showed a neutral loss of 146 amu in agreement with ellagic acid rhamnoside. Compounds **15**, **17**, **25**, and **32** presented a common [M − H]^−^ molecular ion at *m*/*z* 935.3 amu. The neutral loss of 302 amu pointed out to a hexahydroxydiphenoyl (HHDP) group and the loss of 170 amu to a gallate unit. The compounds showed a fragmentation pattern that indicated the presence of two HHDP units and a gallate unit linked to the hexose core. The compounds were tentatively identified as casuarictin/potentillin isomers [19]. Compound **1** showed the characteristic fragmentation pattern of an ellagitannin with two HHDP units. The [M − H]^−^ molecular ion at *m*/*z* 783.3 amu, and MS^2^ fragment ions at *m*/*z* 480.7 and 300.6 allowed the tentative identification of this compound as a pedunculagin isomer [31]. Compound **6** showed a [M − H]^−^ molecular ion at *m*/*z* 633.9 amu and MS^2^ fragment ions at *m*/*z* 462.7 [M – H − galloyl]^−^ and 300.8 amu [M − H − galloyl hexose]^−^. The compound was identified as HHDP-galloyl-hexoside [31]. In Chilean *F. chiloensis* ssp. *chiloensis* f. patagonica, Simirgiotis et al. [26] and Thomas-Valdés et al. [32] reported the presence of 10 ellagitannins, ellagic acid pentoside, ellagic acid rhamnoside, and ellagic acid. Ellagitannins are the main components of strawberries and have been associated with their health-promoting properties [33].

#### 2.1.4. Flavan-3-ols and Proanthocyanidins

Flavan-3-ols and proanthocyanidins were detected in the non-anthocyanin extract of the three Argentinean Patagonia berries species investigated (Table 3). Compound **9** showed a [M − H]^−^ molecular ion at *m*/*z* 289.3 amu and UV_max_ at 280 nm, in agreement with (*epi*)-catechin. Compounds **4**, **7**, **12**, **20,** and **40** showed a common [M − H]^−^ molecular ion at *m*/*z* 577.3 amu and a MS^2^ fragment ion at *m*/*z* 289.1 amu. This fragmentation pattern agreed with a B-type procyanidin dimer [34]. Simirgiotis et al. [26] described the presence of two procyanidin tetramers in Chilean *F. chiloensis* ssp. *chiloensis* f. patagonica. However, no information regarding the presence of proanthocyanidins in Chilean *Berberis* species was found in the literature.

#### 2.1.5. Flavonols

The flavonol composition of the three studied species is depicted in Table 3. The presence of myricetin derivatives was confirmed by the MS^2^ fragment ion at *m*/*z* 316.5 amu and UV_max_ at 365 nm. Compounds **30** and **31** were assigned as myricetin hexosides by the neutral loss of 162 amu, while compound **34** was identified as myricetin rutinoside by the neutral loss of 308 amu. In addition, the presence of dimethylmyricetin hexoside (siringetin hexoside, **61**) was suggested by the MS^2^ fragment ion at *m*/*z* 344.6 amu [30].

Compounds **41**, **42**, **43**, **45**, **47**, **48**, **50**, **52**, **53**, **55,** and **56** showed a MS^2^ fragment ion at *m*/*z* 300.6 amu in agreement with quercetin. The neutral loss of 132 amu for compounds **41**, **42,** and **55** indicated the presence of a pentoside. Compounds **43** and **47** showed a neutral loss of 162 amu in agreement with hexoside, and compound **45** showed a neutral loss of 176 amu, characteristic of a glucuronide. The neutral loss of 146 amu for compound **56** indicated a rhamnose (deoxyhexose), and the neutral loss of 308 amu for compound **48** supported a rutinoside. Compounds **50**, **52,** and **53** presented a common [M − H]^−^ molecular ion at *m*/*z* 505.3 amu, followed by the neutral loss of 204 amu, suggesting the presence of an acetylhexoside, as previously reported in *Berberis* species from the Chilean Patagonia [14]. The compounds were tentatively assigned as quercetin acetylhexosides.

Six kaempferol derivatives were assigned based on the MS^2^ ion at *m*/*z* 285 amu. The hexosides (**54** and **64**), rutinoside (**57**), rhamnoside (**66**), and acetylhexosides (**60** and **68**) were identified by the neutral losses of 162, 308, 146, and 204 amu, respectively. The MS^2^ ion of compounds **58**, **62**, **63**, **65,** and **67** at *m*/*z* 314.7 amu agreed with an isorhamnetin core. The identity of the compounds was established by the neutral losses of hexoside (**62**), rutinoside (**63**), dihexoside rhamnoside (**58**), and acetylhexosides (**65** and **67**), respectively. In Chilean *Berberis microphylla*, Ruiz et al. [14] described the presence of three myricetin, seven quercetin, and five isorhamnetin derivatives. Most of the compounds were hexosides, rutinosides, acetyl hexosides, or rutinoside-hexoside derivatives. A difference of the Argentinean Patagonia *Berberis* samples described in this article with the western Patagonia collections of the same species is the occurrence of the kaempferol derivatives in the eastern Andes populations of Argentina. In Chilean *F. chiloensis* ssp. *chiloensis* f. patagonica, quercetin glucuronide, quercetin pentoside, kaempferol glucuronide, and two kaempferol coumaroyl-hexosides were described by Simirgiotis et al. [26].

### 2.2. Quantification of Main Phenolics

**Figure 6:**
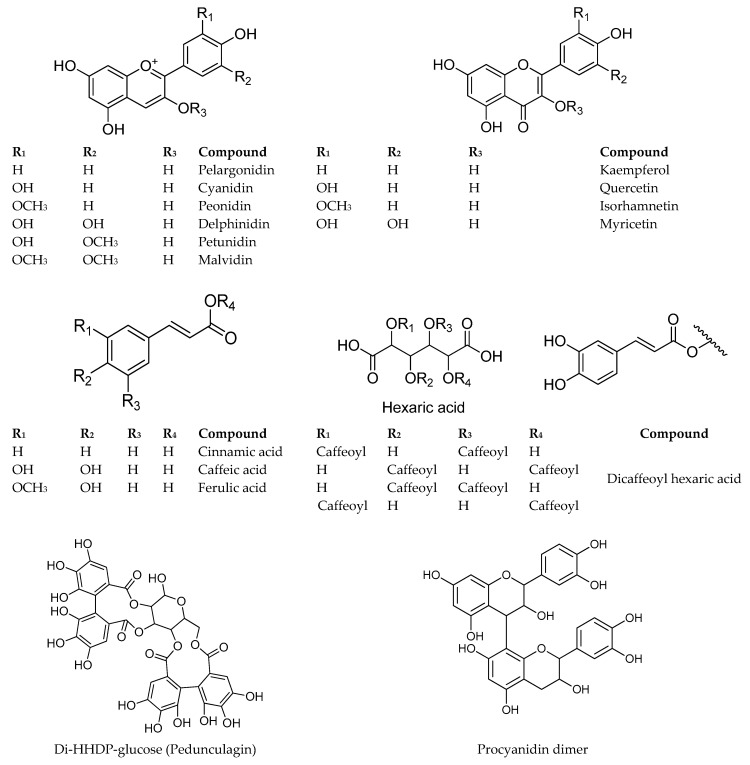
Main group of phenolic compounds occurring in *Berberis* and *Fragaria chiloensis*.

Main group of phenolic compounds occurring in *Berberis* and *Fragaria chiloensis* are shown in Figure 6. The content of individual anthocyanins of the Argentinean Patagonia berries is depicted in Table 4 for *Fragaria chiloensis* sp. *chiloensis* f. patagonica, and in in Table 5 for *Berberis microphylla* and *B. darwinii*. The main anthocyanin in *F. chiloensis* was cyanidin-3-glucoside (**3a**) and ranged from 0.7–7.1 mg/100 g fresh weight (fw), followed by pelargonidin hexoside (**6a**), with contents ranging from 1.7–5.8 mg/100 g fw. In Chilean samples, the main anthocyanins were pelargonidin derivatives, followed by cyanidin derivatives, and the same trend was observed in the commercial *Fragaria* x *ananassa* cv. Chandler [17].

In the Argentinean Patagonia *Berberis microphylla*, the main anthocyanin was delphinidin-3-glucoside (**1a**), with contents ranging from 78.6–621.7 mg/100 g fw, followed by petunidin hexoside (**5a**), ranging from 35.7–363.6 mg/100 g fw. From all the five collection places, the sample from Brazo Rincon showed the lowest content of anthocyanins. This might be explained by the ripening stage of the fruits collected in this location. The total anthocyanin content of other berries has shown to increase during the ripening period, visualized as the fruit skin color becomes darker [35]. In the Chilean samples studied by Ruiz et al. [20], the main anthocyanins of *B. microphylla* were delphinidin-3-glucoside and petunidin-3-glucoside, with contents of 410.6 and 225.6 mg/100 g fw, respectively. In *B. darwinii* the main anthocyanins were also delphinidin-3-glucoside and petunidin hexoside, with contents ranging from 115.3–163.3 and 61.9–83.7 mg/100 g fw, respectively. No information regarding *B. darwinii* fruits from Chile could be found in the literature. Other species, such as *Berberis ilicifolia* and *Berberis empetrifolia*, collected in the Chilean Patagonia, showed the same pattern, with delphinidin-3-glucoside and petunidin-3-glucoside being the main components. The content of delphinidin-3-glucoside and petunidin-3-glucoside was 132.5 mg/100 g fw and 117.3 mg/100 g fw in *B. ilicifolia*, and 234.8 mg/100 g fw and 150.9 mg/100 g fw in *B. empetrifolia*, respectively [20].

In *Fragaria chiloensis*, the main flavonol was quercetin pentoside 2 (**42**) with contents ranging from 6.2–7.1 mg/100 g fw, followed by quercetin pentoside 3 (**55**), ranging from 2.4–2.8 mg/100 g fw. In Chilean *Fragaria* samples, the content of quercetin and kaempferol after acid hydrolysis were reported as 0.6 and 1.1 mg/100 g fw, respectively [26].

In *B. microphylla*, the main flavonols were quercetin rutinoside (rutin) (**48**) and isorhamnetin rutinoside (**63**), with contents ranging from 14.1–53.5 and 4.2–55.7 mg/100 g fw, respectively. In *B. darwinii*, the main flavonol was quercetin acetylhexoside 1 (**52**) (25.2–59.8 mg/100 g fw), followed by quercetin hexoside (**43**) (29.8–56.6 mg/100 g fw). In the Argentinean samples, Arena et al. [7] reported contents of rutin in the range of 0.5–1.0 mg/100 g fw and quercetin 1.9–2.6 mg/100 g fw. The concentration of these flavonols did not show variation in response to the light intensity or fertilization level of the plants [7].

Hydroxycinnamic acids (HCAs) were not detected in the Argentinean Patagonia *Fragaria* samples. However, in Chilean *Fragaria* species, Parra-Palma et al. [36] described the presence of 4-coumaric, ferulic, and cinnamic acids, with concentrations in the mg/kg range. The main HCA in *B. microphylla* was caffeoylquinic acid 3 (**13**), with contents ranging from 31.6–163.7 mg/100 g fw, followed by dicaffeoyl glucaric acid 2 (**14**), ranging from 17.7–56.3 mg/100 g fw. In Chilean *B. microphylla*, the main HCA was 5-caffeoylquinic acid, with contents ranging from 1.4–98.4 mg/100 g fw [21]. In addition, the same authors reported that caffeoylglucaric acids were about 50% of the total HCA content. In *B. darwinii*, the main HCA was also caffeoylquinic acid 3 (**13**), with contents ranging from 100.0–328.3 mg/100 g fw, followed by caffeoylglucaric acid 2 (**5**), with contents between 59.2 and 217.6 mg/100 g fw (Table 5). In Argentinean collections, Arena et al. [7] reported the content of chlorogenic acid, ferulic acid, and gallic acid in *Berberis microphylla* fruits under different light and fertilization conditions. The chlorogenic and ferulic acid contents were in the range of 113.9–130.3 mg/100 g fw, and 4.3–4.9 mg/100 g fw, respectively.

Our results with the Argentinean Patagonia samples showed variation in the phenolic content of *B. microphylla*. The content of delphinidin hexoside (**1a**), dicaffeoyl glucaric acid 2 (**14**), dicaffeoyl glucaric acid 6 (**36**), dicaffeoylquinic acid 7 (**51**), and quercetin rutinoside (**48**) significantly varied among the five collection places (Table 5, *p* < 0.05). This variation could be related to environmental and/or genetic factors of the plant populations [37]. In Argentinean *Berberis* fruits, Arena et al. showed that under field conditions, the light intensity and fertilization of plants increased the photosynthetic rate, soluble solids, sugars, and anthocyanins [7,38].

### 2.3. Antioxidant Activity

The study of the potential antioxidant effects of natural products demands the use of several antioxidant assays. This can be considered as a first approach to an in vivo situation, since different reactive species and mechanisms are involved in oxidative stress.

In the 2,2-diphenyl-1-picrylhydrazyl radical (DPPH) assay of the Argentinean Patagonia samples, the highest scavenging capacity was found in one of the PEEs from *F. chiloensis* collected in Frey, followed by the *B. darwinii* sample from Villa La Angostura (Table 1). In the ferric-reducing antioxidant power (FRAP), trolox equivalent antioxidant activity (TEAC), and cupric-reducing antioxidant power (CUPRAC) assays, the best antioxidant activity was found in both extracts of *B. darwinii*. However, in the oxygen radical absorbance capacity (ORAC) assay, the best antioxidant activities were found in *B. microphylla* fruit extracts (Table 1). Ramirez et al. [15] described the antioxidant capacity of six berries, including *B. microphylla* samples collected in central southern Chile. Their results showed the best radical scavenging capacity in the DPPH assay, and the best reduction power in the FRAP assay for *B. microphylla*. In the study of Ruiz et al. [20] with Chilean Patagonian berries, the best antioxidant capacity in the TEAC assay was found in *B. microphylla* samples. Ruiz et al. [14] compared the antioxidant capacity of *B. microphylla* with the popular maqui berry (*Aristotelia chilensis*) and showed that the maqui berry had the highest antioxidant capacity by means of TEAC assay. The highest ORAC values among 120 Chilean fruit species analyzed were found for *B. microphylla*, *A. chilensis*, and *Ugni molinae* [6]. Thomas-Valdés et al. [32] described the antioxidant activity of Chilean *Fragaria chiloensis* fruits by means of DPPH, FRAP, TEAC, and superoxide anion scavenging. Similar values were observed in the DPPH assay, while in the FRAP and TEAC assays, the Chilean samples presented higher values than the Argentinean collections. Arena et al. [7] showed that the exposure of *Berberis microphylla* plants to high light intensity was related to higher antioxidant capacities of the fruits, measured by DPPH and FRAP assay. The use of fertilization also increased the antioxidant power of *B. microphylla* fruits by 5% [7].

The Pearson’s correlation coefficient showed that the individual anthocyanins quantified presented strong correlations (*p* < 0.05) with the antioxidant activity (Table 6), except for compounds **8a** and **11a**. For the HCAs, the content of caffeoyl hexaric acid isomer 2 (**5**), caffeoylquinic acid isomer 3 (**16**), and feruloylquinic acid isomer 1 (**18**) also demonstrated strong correlations with all the antioxidant assays carried out (Table 6). Regarding flavonols, the content of quercetin hexoside 1 (**43**), quercetin rutinoside (**48**), and quercetin rhamnoside (**56**) showed strong correlations with all the antioxidant assays (*p* < 0.01), while the content of isorhamnetin acetylhexoside isomer 2 (**67**) presented significant correlations with all the assays, except in the TEAC method (Table 6).

### 2.4. Inhibition of Metabolic Syndrome-Associated Enzymes

The inhibition of α-amylase and α-glucosidase is a therapeutic strategy for the control of post-prandial hyperglycemia. Polyphenols present in food and beverages can easily reach mM concentrations in the gut, even when diluted with other foods and digestive fluids. They can interact with these digestive enzymes, changing the glycemic responses by inhibiting digestion of carbohydrates [39]. All the Argentinean Patagonia samples investigated in this work are α-glucosidase inhibitors (Table 1). The positive control acarbose showed an IC_50_ value of 137.73 µg/mL, while the IC_50_ values of the samples ranged from 0.14–1.19 µg PEE/mL. The IC_50_ values obtained against α-glucosidase showed a significant Pearson’s correlation with the content of cyanidin-3-glucoside (**3a**), caffeoylglucaric acid isomer 2 (**5**), caffeoylquinic acid isomer 3 (**16**), feruloylquinic acid isomer 1 (**18**), and isorhamnetin acetylhexoside isomer 2 (**67**) (*p* < 0.01) (Table 6).

Under our experimental conditions, none of the samples inhibited α-amylase, while the positive control acarbose showed an IC_50_ value of 28.5 µg/mL. In vivo studies have demonstrated the preventive role of berries against metabolic syndrome and type 2 diabetes. For example, the supplementation of the human diet with two cups (150 g) of lingonberries (*Vaccinum vitis-idea*) or blackcurrants (*Ribes nigrum*) reduced the postprandial glucose and insulin levels in the first 30 min after the intake [40]. Reyes-Farias et al. [41] showed that *Berberis microphylla* extracts improved glucose uptake in 3T3-L1 mouse adipocytes pre-treated with lipopolysaccharides (LPS). This was explained by the authors as an insulin-sensitization feature of the *B. microphylla* extract and was associated to the high content of anthocyanins of this fruit.

Pancreatic lipase splits triglycerides into absorbable glycerol and fatty acids. Its inhibition by drugs such as Orlistat has been employed to treat obesity. Several studies have shown that the polyphenols present in beverages and fruits have inhibitory effects in lipase that could be relevant to regulate fat digestion, and thus the energy intake and obesity [33]. Under our experimental conditions, only the PEEs from *F. chiloensis* inhibited this enzyme, with IC_50_ values of 38.3 ± 1.6 and 41.4 ± 0.7 µg PEE/mL for the Frey and Arroyo Llodcondo samples, respectively. The PEEs of the Chilean strawberries *F. chiloensis* ssp. *chiloensis* f. chiloensis and *F. chiloensis* ssp. *chiloensis* f. patagonica inhibited pancreatic lipase by 70% and 41% at 50 µg/mL, respectively [26,34]. The inhibitory capacity of both fruits’ PEEs withstands in some extent a simulated gastrointestinal digestion model [32,42]. The acetone fruit extract from the commercial strawberry *F*. x *ananassa* inhibited pancreatic lipase, α-amylase, and α-glucosidase with IC_50_ values of 73.04, 18.18, and 156.36 mg fresh fruit/mL, respectively [43]. McDougall et al. [33] showed that lipase activity was effectively inhibited by the ellagitannins present in cloudberry, raspberry, and strawberry extracts, with a partial contribution of proanthocyanidins. Considering our results, we can also hypothesize that the presence of ellagitannins in *F. chiloensis* may be responsible for this inhibitory activity. However, the quantification of these compounds was not possible in our samples because of the lack of authentic standards. Future studies are needed to determine the Pearson’s coefficient of ellagitannins with this inhibitory activity.

## 3. Materials and Methods

### 3.1. Chemicals

From Sigma-Aldrich (St. Louis, MO, USA): Amberlite XAD7^®^, α-amylase from porcine pancreas (A3176; EC 3.2.1.1), α-glucosidase from *Saccharomyces cerevisiae* (G5003; EC 3.2.1.20), DPPH (2,2-diphenyl-1-picrylhydrazyl radical), 3,5-dinitrosalicylic acid, catechin, CuCl_2_, lipase from porcine pancreas type II (L-3126; EC 3.1.1.3), 4-nitrophenyl-α-D-glucopyranoside, *p*-nitrophenyl palmitate, quercetin, sodium acetate, starch, TPTZ (2,4,6-tri(2-pyridyl)1,3,5-triazine), and triton X-100. From Merck (Darmstadt, Germany): ABTS (2,2′-azino-bis(3-ethylbenzothiazoline-6-sulphonic acid), trolox (6-hydroxy-2,5,7,8-tetramethylchroman-2-carboxylic acid), FeCl_3_ × 6H_2_O, neocuproin, potassium sodium tartrate, and HPLC-grade methanol. Ammonium acetate was from JT Baker (Xalostoc, Mexico). The following standards were from PhytoLab (Vestenbergsgreuth, Germany): malvidin-3-glucoside chloride (89728, 99.3% purity), quercetin-3-glucoside (89230, 99.1% purity), and chlorogenic acid (89175, 98.9% purity). Orlistat was from Laboratorio Chile (Santiago, Chile).

### 3.2. Sample Collection

Ripe fruits from the selected species were collected during the summer season (December–February) of 2016–2017 in the Argentinean Patagonia, at the Nahuel Huapi National Park and surroundings. Fruits from *Berberis microphylla* were from: (1) Brazo Rincón (40°72′07″ S; 71°79′76″ W, voucher specimen: 700–708MC), (2) Aeropuerto (41°14′51″ S; 71°17′67″ W, voucher specimen 620–636MC), (3) Villa La Angostura (40°49′50″ S; 71°64′31″ W, voucher specimen 720–727MC), (4) Cuyín Manzano (40°76′57″ S; 71°17′37″ W, voucher specimen 680–688MC), and (5) Llanquín (40°76′57″ S; 71°17′37″ W, voucher specimen 660–671MC). *Berberis darwinii* fruits were collected at: (1) Brazo Rincón (40°72′17″ S; 71°80′20″ W, voucher specimen 710–718MC) and (2) Villa La Angostura (40°50′51″ S; 71°64′93″ W, voucher specimen 730–735MC). The fruits from *Fragaria chiloensis* ssp. *chiloensis* f. patagonica were collected at (1) Arroyo Llodcondo (41°14′41″ S; 71°31′38″ W, voucher specimen 600MC) and (2) Frey (41°17′80″ S; 71°44′17″ W, voucher specimen 640MC). The plant material was identified by Dr. Ana Ladio and Dr. Melina Chamorro. Voucher herbarium specimens were deposited at the Herbario del Grupo de Etnobiología del INIBIOMA, Laboratorio Ecotono, Bariloche, Argentina.

Fruits were transported to the laboratory and frozen at −80 °C. Samples were then freeze-dried (Biobase Bk FD 10, Biobase Biodustry, Shandong, China), and the water content was determined by weight difference (Table 1). The weight of the freeze-dried fruits varied from 11–56 g. The fruits were powdered in a Waring blender (Thomas TH-501V, Thomas Elektrogeräte, Shanghai, China) to a final particle size of 0.35 mm (45 mesh) and extracted four times using MeOH:formic acid (99:1 *v*/*v*) in a 1:5 w/v ratio (total volume of extraction ranged from 55–250 mL). Extraction of phenolic compounds was enhanced using a sonicator bath at 35 kHz (Elma Transsonic 700, Elma GmbH & Co. KG, Singen, Germany) for 15 min each time. The extracts were dried under reduced pressure at 35 °C in a rotary evaporator (Laborota 4001, Heildolph, Schwabach, Germany). Then, the methanol extract was dissolved in 1 L of water, sonicated to increase the solubility, and phenolics were retained in an Amberlite XAD7 column. The column was washed with 2 L of water and then compounds were desorbed with 2 L of MeOH:formic acid (99:1, *v*/*v*). The polyphenol-enriched extract (PEE) obtained was evaporated under reduced pressure in the rotary evaporator and then freeze dried. The yield of extraction was calculated as the percent of PEE obtained from 100 g of fresh fruit.

### 3.3. Anthocyanin and Non-Anthocyanin Polyphenol Fractionation

The fractionation of anthocyanins and non-anthocyanin polyphenols was carried out using a solid phase extraction (SPE) cartridge Bond Elut Plexa PCX 6mL (Agilent, Santa Clara, CA, USA) [44]. Briefly, the cartridge was preconditioned with 5 mL of MeOH and 5 mL of ultrapure water. Samples were dissolved in MeOH:H_2_O:formic acid (50:48.5:1.5) at a concentration of 1 mg/mL, sonicated for 6 min at 30 °C, and filtered. Then, 3 mL of this solution was reduced to 1.5 mL in a rotary evaporator and 1.5 mL of HCl 0.1N was added. After this, the samples were passed through the cartridge at a flow rate of 0.2 mL/min. The PCX cartridge was washed with 5 mL of 0.1 N HCl, 5 mL of ultrapure water, and then completely dried. The non-anthocyanin polyphenols were recovered with 6 mL of 96% ethanol (EtOH). Anthocyanins were desorbed with 2% HCl in MeOH:H_2_O (8:2, *v*/*v*). Anthocyanins and non-anthocyanin polyphenols were dried under reduced pressure at 30 °C and freeze dried for the subsequent HPLC analyses.

### 3.4. HPLC-DAD-ESI-MS^n^ Analysis

HPLC analyses were performed in an Agilent Series 1100 HPLC system equipped with a G1311 quaternary pump, a G1315B diode array detector, a G1322A degasser, G1313A autosampler, and a liquid chromatography mass selective detector (LC/MSD) Trap VL G-2445 electrospray ionization mass spectrometry (ESI-MS^n^) detector. The control of the system and data analysis was achieved using ChemStation software (Agilent Technologies, Waldbronn, Germany). The separation was carried out using a Zorbax Eclipse XDB C18 column (3.5μm, 150 × 2.1 mm) (Agilent, Germany). The solvent systems were A (H_2_O-formic acid-acetonitrile (ACN), 88.5:8.5:3, *v*/*v*/*v*); B (H_2_O-formic acid-ACN; 41.5:8.5:50, *v*/*v*/*v*); and C (H_2_O-formic acid-MeOH, 1.5:8.5:90, *v*/*v*/*v*). A flow rate of 0.19 mL/min was used and temperature was set at 40 °C, with an equilibration time of 8 min in the initial conditions before the next injection. The peaks were numbered according to the retention time and followed by the letter “a” for anthocyanins, and no letter for other compounds.

Anthocyanins were analyzed using the following gradient only with solvents A and B: *t* = 0 min, 94% A, 6% B; *t* = 10 min, 70% A, 30% B; *t* = 34 min, 0% A, 100% B; *t* = 36 min, 0% A, 100% B; *t* = 42 min, 94% A, 6% B. For the ESI-MS^n^ analysis of anthocyanins in the positive mode, nitrogen was used as the nebulizer gas at 50 psi, 325 °C, and at a flow rate of 8 L/min. Electrospray needle, −2500 V; skimmer 1, 19.2 V; skimmer 2, 5.7 V; capillary exit offset 1, 33.0 V; capillary exit offset 2, 52.1 V. The scan mode was performed at a speed of 13,000 *m*/*z*/s, in the range of 50–1200 *m*/*z*.

The non-anthocyanin polyphenol analysis was carried out using the following gradient: *t* = 0 min, 98% A, 2% B and 0% C; *t* = 8 min, 96% A, 4% B and 0% C; *t* = 37 min, 70% A, 17% B and 13% C; *t* = 51 min, 50% A, 30% B and 20% C; *t* = 51.5 min, 30% A, 40% B and 30% C, *t* = 56 min, 0% A, 50% B and 50% C; *t* = 57 min, 0% A, 50% B and 50% C; *t* = 64 min, 98% A 2% B and 0% C. For the ESI-MS^n^ analysis in the negative mode, nitrogen was used as the nebulizer gas at 40 psi, 350 °C, and at a flow rate of 8 L/min. Electrospray needle, 3500 V; skimmer 1, 20.3 V; skimmer 2, 6.0 V; capillary exit offset 1, 68.2 V; capillary exit offset 2, 88.5 V. The scan mode was performed at a speed of 13,000 *m*/*z*/s, in the range of 50–1000 *m*/*z*.

Quantification was carried out using external calibration curves. The analytical parameters were calculated in agreement to the International Conference on Harmonisation (ICH) guidelines [45]. Five-point calibration curves were prepared in triplicate using the commercial standards: malvidin-3-glucoside (8–420 mg/L, r^2^: 0.9989) for anthocyanins, quercetin-3-glucoside (10–100 mg/L, r^2^: 0.9996) for flavonols, and chlorogenic acid (1–100 mg/L, r^2^: 0.9996) for hydroxycinnamic acids. Integrated area under the curve (AUC) was calculated for peaks observed at 520 nm, 360 nm, and 320 nm for anthocyanins, flavonols, and HCAs, respectively. Results were expressed as mg/100 g fresh fruit.

### 3.5. Antioxidant Capacity Assays

The antioxidant capacity of the samples was evaluated by means of the following assays: discoloration of the DPPH and ABTS^•+^ radical (TEAC), ferric- and cupric-reducing antioxidant power (FRAP and CUPRAC), and the oxygen radical absorbance capacity (ORAC). Quercetin was used as the reference compound in all the antioxidant assays.

The DPPH assay was carried out according to Bondet et al. [46], with slight modifications. Briefly, a stock solution of DPPH radical (20 mg/L) was prepared in MeOH and stored in the dark. Samples were prepared in MeOH at final concentrations ranging from 0–100 µg PEE/mL. The discoloration of the radical after 5 min of incubation was measured at 517 nm in a microplate reader (BioTek ELX800, Winooski, VT, USA). The results were expressed as the concentration of extract that scavenged the free radical by 50% (SC_50_, µg PEE/mL). The FRAP and CUPRAC assays were carried out as previously described [19]. Briefly, the FRAP solution was prepared by mixing 300 mM acetate buffer (pH 3.6) with 10 mM TPTZ prepared in 40 mM HCl and 20 mM FeCl3 in a 10:1:1 *v*/*v*/*v* proportion. The sample was prepared in MeOH at final concentrations ranging from 0–100 µg PEE/mL. The reduction of the ferric ion complex was read after 30 min at 593 nm in a spectrophotometer Genesys 10UV (Thermo Spectronic, Waltham, MA, USA). The CUPRAC assay was carried by mixing 1 M ammonium acetate (pH 7.0) with 0.01 M CuCl2 and 7.5 mM neocuproin solution in a 1:1:1 proportion. Then, the sample (0–100 µg PEE/mL) was added and the reduction of the cupric ion was measured at 450 nm after 30 min incubation in the dark. In the FRAP and CUPRAC assays, the results were expressed as μmol Trolox equivalents (TE)/g of PEE. The scavenging of the ABTS^•+^ radical (TEAC) was carried out according to Nenadis et al. [47]. Briefly, the ABTS^•+^ radical was prepared by mixing 88 µL of 140 mM sodium persulfate with 5 mL of 7.5 mM ABTS solution. The mixture was incubated overnight at room temperature. The following day, the ABTS^•+^ radical solution was diluted with MeOH to final absorbance of 0.700 ± 0.005 at 734 nm. Samples were prepared in MeOH at concentrations ranging from 50–300 µg PEE/mL. Thirty µL of each dilution was mixed with 2.870 mL of the ABTS^•+^ radical solution and incubated for 6 min. Final absorbance was measured and results were expressed as μM TE/g of PEE. The ORAC assay was carried out according to Ou et al. [48]. Briefly, a 110 nM fluorescein working solution was prepared in 75 mM sodium phosphate buffer. A 152.6 mM 2,2’-azobis (2-methhylpropionamidine) dihydrochloride (AAPH) solution was prepared in the same buffer and incubated for 30 min at 37 °C right before mixing with the samples. Samples (5–25 µg PEE/mL) and Trolox (0–50 µM) were prepared in the same buffer. The assay mixture consisted of 150 µL fluorescein + 25 µL sample or standard + 25 µL AAPH. Fluorescence was read at λ_ex_ 485/λ_em_ 528 nm every min for 90 min in a Synergy HT multidetection microplate reader (Bio-Tek Instruments Inc., Winooski, VT, USA). Results were expressed as μmol TE/g PEE. All samples were assayed in triplicate and results were presented as mean values ± SD.

### 3.6. Inhibition of Metabolic Syndrome-Associated Enzymes

The capacity of the samples to in vitro inhibit carbohydrate and lipid metabolism was evaluated by means of the following assays: inhibition of α-glucosidase, α-amylase, and pancreatic lipase.

The α-glucosidase inhibition assay was carried out as described by Jiménez-Aspee et al. [23]. The reaction mixture contained sodium phosphate buffer (200 mM, pH 6.6), sample (0.1–100 μg PEE/mL), and α-glucosidase (0.25 U/L). After 15 min of pre-incubation at 37 °C, the reaction was started by adding *p*-nitrophenyl-α-d-glucopyranoside (5mM). The mixture was further incubated for 15 min at 37 °C. The reaction was stopped by adding 0.2 M sodium carbonate. Absorbance was read at 415 nm in a microplate reader (ELx800, Biotek, Winooski, VT, USA). All samples were assayed in triplicate and the results were expressed as IC_50_ values (μg PEE/mL). Acarbose was used as the positive control [23].

The α-amylase inhibition assay was carried out as described by Jiménez-Aspee et al. [23]. Briefly, the samples (0.1–100 μg/mL) were incubated with 1% starch for 5 min at 37 °C. Then, the α-amylase solution (8 U/mL) was added and incubated for a further 20 min. After the incubation, 400 µL of the color reagent (96 mM 3,5-dinitrosalicylic acid, 5.31 M sodium potassium tartrate in 2 M NaOH) were added, and the mixture was boiled for 15 min. Absorbance was measured in a microplate reader at 550 nm (Biotek Elx800). Acarbose was used as the positive control [23]. All samples were assayed in quadruplicate and the results were expressed as IC_50_ values (μg PEE/mL).

The lipase inhibition assay was carried out as described by McDougall et al. [33]. Briefly, the enzyme was re-suspended in ultrapure water (20 mg/mL) and centrifuged at 8000× *g* at 4 °C for 10 min to recover the supernatant for the assay. The substrate was prepared with *p*-nitrophenyl palmitate (0.08% *w*/*v*), 5mM sodium acetate buffer (pH 5.0), and 1% Triton X-100. The assay mixture was 100 mM Tris buffer (pH 8.2), extracts, lipase, and substrate solution. The mixture was incubated for 2 h at 37 °C and absorbance was read at 400 nm in a microplate reader (Biotek ELx800). All samples were assayed in sextuplicate at 50 µg/mL as the maximum concentration. Orlistat^®^ was used as the reference compound [23]. Results were expressed as IC_50_ values (μg PEE/mL).

### 3.7. Statistical Analyses

Statistical analyses were carried out using SPSS 14.0 software (IBM, Armonk, NY, USA). Significant differences among the *Berberis microphylla* samples were determined by one-way analysis of variance (ANOVA), followed by Tukey’s multiple comparison test (*p* < 0.05). This analysis is not possible to carry out with less than three samples, which was the case for the other studied species. Pearson’s correlation coefficients were calculated to determine the relationship between antioxidant activity and the content of main compounds.

## 4. Conclusions

The phenolic profiles, antioxidant activity, and inhibitory effect towards the enzymes α-glucosidase, α-amylase, and pancreatic lipase of three Argentinean Patagonia berries were investigated. The most complex polyphenol profile among the studied species was found in the *Berberis* samples, with 10 anthocyanins, 27 HCAs, 3 proanthocyanidins, 2 flavan-3-ol, and 22 flavonols. *Fragaria* presented a simpler profile, including four anthocyanins, nine ellagitannins, two proanthocyanidin dimers, one flavan-3-ol, and five flavonols. The composition of the Argentinean Patagonia samples showed as main compounds the same constituents of those in the western Patagonia collections, but differed in minor metabolites, showing different oxidation and/or glycosylation patterns. The proanthocyanidin profile of the Argentinean Patagonia *Berberis* species has been not described so far in Chilean collections. The *Berberis* samples showed the best antioxidant capacity, in agreement with the results reported for Chilean collections. Regarding the inhibition of the metabolic syndrome-associated enzymes, the *Fragaria* samples showed potential to modulate carbohydrate and fat metabolism, as observed for the Chilean/western Patagonia samples. The weakness of our work relies on the small sample numbers for *B. darwinii* and *F. chiloensis*. In addition, because of the low amount of starting material, compound isolation was not possible and the identification was only based in the tentative assignment by mass spectrometry. On the other hand, the strength of our work is that this is the first work about the secondary metabolite content and composition of some Argentinean Patagonian berries and contributes to the knowledge of the chemistry of genus *Berberis*. In addition, our results provide evidence on the berry constituents, and highlight some of their potential health-promoting properties. More studies are needed to select high productive individuals for plant-breeding programs and to promote production of these species in the Argentinean and Chilean Patagonia.

## Figures and Tables

**Table 1:** Antioxidant capacity and inhibition of metabolic syndrome-associated enzymes by the phenolic enriched extracts (PEE) of wild berries from Argentinean Patagonia. DPPH = 2,2-diphenyl-1-picrylhydrazyl radical, FRAP = ferric-reducing antioxidant power, CUPRAC = cupric-reducing antioxidant power, ORAC = oxygen radical absorbance capacity, TEAC = Trolox equivalent antioxidant capacity.

Samples	Yield of Extraction (%*w*/*w*)	Moisture (%*w*/*w*)	DPPH (SC_50_, µg/mL)	FRAP (µmol TE/g PEE)	TEAC (µM TE/g PEE)	CUPRAC (µmol TE/g PEE)	ORAC (µmol TE/g PEE)	α-glucosidase (IC_50_, µg/mL)
***Berberis microphylla***								
Brazo Rincón	9.2	72.1	16.0 ± 0.4 ^a^	1915.0 ± 61.0 ^a^	Inactive	2876.3 ± 78.7 ^a^	843.8 ± 43.0 ^a^	0.5 ± 0.0 ^a^
Aeropuerto	8.4	54.5	10.4 ± 0.3 ^b^	2656.6 ± 43.7 ^b^	1938.8 ± 58.8 ^a^	3355.0 ± 103.7 ^b^	3020.0 ± 99.3 ^b^	0.4 ± 0.0 ^d^
Villa La Angostura	9.9	58.4	10.4 ± 0.4 ^b^	2579.6 ± 45.5 ^b^	1565.8 ± 33.3 ^b^	3470.7 ± 19.0 ^b^	2800.8 ± 90.7 ^b^	0.3 ± 0.0 ^b^
Cuyín Manzano	7.3	53.0	10.9 ± 0.4 ^b^	2578.0 ± 36.8 ^b^	1760.2 ± 44.3 ^c^	3318.0 ± 73.2 ^b^	3299.8 ± 69.7 ^c^	0.1 ± 0.0 ^c^
Llanquín	8.7	57.5	15.3 ± 0.4 ^a^	2009.7 ± 46.3 ^a^	1748.3 ± 46.2 ^c^	3015.4 ± 96.4 ^a^	2928.0 ± 94.1 ^b^	0.4 ± 0.0 ^d^
***Berberis darwinii***								
Brazo Rincón	11.4	74.6	17.3 ± 0.2	2770.6 ± 76.8	2012.5 ± 39.8	4588.9 ± 157.4	3032.3 ± 60.3	0.5 ± 0.0
Villa La Angostura	12.3	72.8	8.8 ± 0.2	3014.6 ± 63.0	2280.0 ± 44.8	4937.6 ± 89.4	2160.3 ± 164.4	0.8 ± 0.0
***Fragaria chiloensis***								
Arroyo Llodcondo	6.1	83.1	10.1 ± 0.3	1064.8 ± 15.4	1484.8 ± 29.1	2292.8 ± 27.3	772.5 ± 0.9	0.3 ± 0.0
Frey	5.3	82.0	8.4 ± 0.1	1722.4 ± 36.8	1234.8 ± 40.1	2744.4 ± 59.7	811.0 ± 2.6	1.2 ± 0.2
***Reference compounds***								
Quercetin	-	-	7.8 ± 0.3	1077.2 ± 16.4	8157.9 ± 22.1	27,526.1 ± 97.5	22,561.6 ± 808.8	-
Acarbose	-	-	-	-	-	-	-	137.7 ± 1.3
Orlistat	-	-	-	-	-	-	-	-

-: not determined. Different letters (^a–e^) in the same column show significant differences among each determination according to Tukey’s test (*p* < 0.05).

**Table 2:** Characterization and distribution of anthocyanins in Argentinean Patagonia *Berberis micropylla*, *B. darwinii*, and *F. chiloensis* berries by HPLC-DAD-ESI-MS^n^.

Peak	Rt (min)	[M + H]^+^	MS^2^	λ_max_ (nm)	Tentative Identification	Detected in		
						*B. microphylla*	*B. darwinii*	*F. chiloensis*
**1a**	**6.7**	**465.3**	**302.9 (100)**	**523**	Delphinidin-3-*O*-glucoside *	x	x	
**2a**	8.2	611.4	302.9 (100)	525	Delphinidin rutinoside	x	x	
**3a**	9.7	449.1	286.9 (100)	516	Cyanidin-3-*O*-glucoside *	x	x	x
**4a**	10.9	595.4	286.9 (100)	521	Cyanidin-3-*O*-rutinoside *	x	x	
**5a**	11.3	479.3	316.8 (100)	525	Petunidin hexoside	x	x	
**6a**	11.6	433.3	271.1 (100)	502	Pelargonidin hexoside			x
**7a**	12.1	625.5	316.9 (100)	527	Petunidin rutinoside	x	x	
**8a**	13.1	463.3	300.9 (100)	518	Peonidin hexoside	x	x	
**9a**	13.8	609.6	300.9 (100)	526	Peonidin rutinoside	x	x	
**10a**	13.9	535.1	286.9 (100)	517	Cyanidin manlonyl hexoside			x
**11a**	14.1	493.3	331.0 (100)	527	Malvidin-3-*O*-glucoside *	x	x	
**12a**	14.6	639.4	331.0 (100)	530	Malvidin rutinoside	x	x	
**13a**	15.5	519.1	271.1 (100)	505	Pelargonidin-malonyl hexoside			x

* Identity confirmed by co-injection with authentic standard. MS^2^: tandem mass spectrometry.

**Table 3:** Characterization and distribution of phenolic compounds in wild Argentinean Patagonia *Berberis microphylla*, *B. darwinii*, and *F. chiloensis* berries by HPLC-DAD-ESI-MS^n^.

Peak	Rt (min)	[M − H]^−^	MS^2^	λ_max_ (nm)	Tentative Identification	Detected in		
						*B. microphylla*	*B. darwinii*	*F. chiloensis*
**1**	**2.8**	**783.3**	**480.8 (41), 300.7 (100)**	**280**	Di-HHDP-glucose (Pedunculagin)			x
**2**	3.0	371.0	208.6 (100), 190.7 (100)	326	Caffeoylhexaric acid isomer1	x	x	
**3**	3.6	353.3	190.6 (100), 178.9 (26), 134.9 (7)	328	5-Caffeoylquinic acid *	x	x	
**4**	3.7	577.3	558.7 (18), 450.7 (43) 424.8 (100), 289.1 (16)	274	Procyanidin B-type dimer 1			x
**5**	3.8	370.9	208.6 (100), 190.7 (26)	327	Caffeoylhexaric acid isomer 2	x	x	
**6**	4.0	633.9	450.7 (6), 300.7 (100)	270	HHDP-galloyl-hexose 1			x
**7**	4.2	577.4	450.6 (41), 424.7 (100), 288.7 (36)	280	Procyanidin B-type dimer 2			x
**8**	4.5	353.2	190.6 (100), 178.8 (5), 128.7 (3)	329	3-Caffeoylquinic acid *	x	x	
**9**	5.1	289.3	244.7 (100), 204.7 (41), 124.7 (7)	282	(*epi*)-catechin		x	x
**10**	5.3	353.3	190.6 (100)	320	Caffeoylquinic acid isomer 1	x	x	
**11**	5.7	337.3	162.6 (100), 118.8 (5)	330	3-*p*-Coumaroylquinic acid		x	
**12**	6.1	577.2	558.8 (14), 450.7 (35), 424.9 (100), 407.4 (58), 289.2 (11)	281	Procyanidin B-type dimer 3	x	x	
**13**	6.5–6.9	352.9	190.6 (100)	324	Caffeoylquinic acid isomer 2	x	x	
**14**	7.6	533.1	370.6 (100), 208.7 (12)	324	Dicaffeoylhexaric acid isomer 1	x		
**15**	7.7	935.0	632.9 (100), 300.8 (25)	270	Casuarictin/Potentillin isomer 1			x
**16**	8.6	353.3	190.7 (100)	330	Caffeoylquinic acid isomer 3	x	x	
**17**	9.0	934.9	632.7 (100), 450.9 (7), 300.8 (25)	270	Casuarictin/Potentillin isomer 2			x
**18**	9.3	367.3	179.7 (100)	328	Feruloylquinic acid isomer 1	x	x	
**19**	9.4	533.5	370.7 (100), 208.7 (18)	326	Dicaffeoylhexaric acid isomer 2	x	x	
**20**	10.5	577.0	558.7 (100), 450.7 (35) 424.7 (100), 406.9 (62) 288.7 (21)	280	Procyanidin B-type dimer 4		x	
**21**	11.2	367.2	192.6 (22), 178.7 (100) 134.9 (32)	330	Feruloylquinic acid isomer 2		x	
**22**	12.0	337.4	190.7 (100)	330	5-*p*-Coumaroylquinic acid		x	
**23**	12.2	353.1	190.7 (100)	320	Caffeoylquinic acid isomer 4		x	
**24**	13.9	533.1	370.6 (100), 208.7 (100)	326	Dicaffeoylhexaric acid isomer 3	x	x	
**25**	16.7	935.3	632.9 (45), 300.6 (100)	270	Casuarictin/Potentillin isomer 3			x
**26**	16.9	515.1	353.0 (100), 190.6 (41)	325	Dicaffeoylquinic acid isomer 1	x		
**27**	17.2	463.0	300.5 (100)	350	Ellagic acid hexoside			x
**28**	18.1	367.6	178.6 (100), 134.8 (31)	330	Feruloylquinic acid isomer 3	x	x	
**29**	18.6	533.4	370.6 (100), 208.6 (19)	328	Dicaffeoylhexaric acid isomer 4	x		
**30**	18.8	479.3	316.7 (100)	360	Myricetin hexoside isomer 1		x	
**31**	20.6	479.5	316.7 (100)	365	Myricetin hexoside isomer 2	x		
**32**	20.6	934.5	915.0 (68), 632.9 (35), 300.7 (42)	280	Casuarictin/Potentillin isomer 4			x
**33**	20.7	533.1	370.6 (100), 208.7 (17)	330	Dicaffeoylhexaric acid isomer 5	x	x	
**34**	21.2	625.3	316.5 (100)	360	Myricetin rutinoside	x	x	
**35**	23.3	367.2	192.6 (22), 178.7 (100) 134.9 (32)	320	Feruloylquinic acid isomer 4		x	
**36**	23.7	533.1	370.6 (100), 208.7 (42)	332	Dicaffeoylhexaric acid isomer 6	x	x	
**37**	24.8	515.1	353.0 (100), 190.6 (42)	330	Dicaffeoylquinic acid isomer 2		x	
**38**	25.0	367.1	178.6 (100), 134.7 (31)	326	Feruloylquinic acid isomer 5	x	x	
**39**	25.1	435.0	301.3 (100)	350	Ellagic acid pentoside			x
**40**	25.2	577.0	424.7 (100), 406.9 (62) 288.7 (21)	280	Procyanidin B-type dimer 3		x	
**41**	25.4	435.0	301.0 (100)	367	Quercetin pentoside isomer 1			x
**42**	26.0	435.1	301.3 (100)	361	Quercetin pentoside isomer 2			x
**43**	26.8	463.4	301.2 (100)	351	Quercetin hexoside isomer 1	x	x	
**44**	27.1	533.0	370.5 (100)	340	Dicaffeoylhexaric acid isomer 7		x	
**45**	27.4	477.3	300.6 (100)	350	Quercetin glucuronide		x	x
**46**	28.0	447.1	300.6 (100)	367	Ellagic acid rhamnoside			x
**47**	28.5	463.0	300.6 (100)	354	Quercetin hexoside isomer 2	x	x	x
**48**	29.0	609.3	300.6 (100)	352	Quercetin rutinoside	x	x	
**49**	30.0	533.3	370.6 (100), 208.6 (43)	330	Dicaffeoylhexaric acid isomer 8		x	
**50**	30.4	505.6	300.6 (100)	354	Quercetin acetylhexoside isomer 1	x		
**51**	30.6	515.4	352.7 (100), 190.7 (22)	327	Dicaffeoylquinic acid isomer 3	x	x	
**52**	31.1	505.3	300.6 (100)	353	Quercetin acetylhexoside isomer 2	x	x	
**53**	31.7	505.3	300.6 (100)	352	Quercetin acetylhexoside isomer 3	x	x	
**54**	32.2	447.4	284.8 (100)	340	Kampferol hexoside	x		
**55**	32.2	433.4	300.6 (100)	350	Quercetin pentoside isomer 3			x
**56**	33.8	447.3	300.6 (100)	356	Quercetin rhamnoside	x	x	
**57**	35.4	593.4	284.6 (100)	340	Kaempferol rutinoside	x	x	
**58**	35.7	785.5	314.6 (100)	354	Isorhamnetin rutinoside hexoside	x	x	
**59**	36.1	515.3	352.7 (100)	326	Dicaffeoylquinic acid isomer 4	x	x	
**60**	36.3	489.4	284.6 (100)	340	Kaempferol acetylhexoside		x	
**61**	36.5	507.1	344.6 (100)	320	Siringetin hexoside		x	
**62**	36.6	477.3	315.7 (100)	345	Isorhamnetin hexoside	x		
**63**	38.3	623.4	315.7 (100)	355	Isorhamnetin rutinoside	x	x	
**64**	39.5	447.5	284.6 (100)	340	Kaempferol hexoside	x		
**65**	40.5	519.3	315.6 (100)	355	Isorhamnetin acetylhexoside isomer 1	x		
**66**	41.0	431.4	284.7 (100)	340	Kaempferol rhamnoside	x	x	
**67**	42.3	519.6	315.6 (100)	340	Isorhamnetin acetylhexoside isomer 2	x		
**68**	44.2	489.7	284.8 (100)	340	Kaempferol acetylhexoside	x		

* Identity confirmed by co-injection with authentic standard.

**Table 4:** Anthocyanins and flavonols content of wild *Fragaria chiloensis* from the Argentinean Patagonia. Data are expressed as mg/100 g fw.

Compounds	*Fragaria chiloensis*	
	Arroyo Llodcondo	Frey
***Anthocyanins***		
Cyanidin-3-glucoside (**3a**)	7.1 ± 0.2	0.7 ± 0.1
Pelargonidin hexoside (**6a**)	5.8 ± 0.1	1.7 ± 0.0
Cyanidin-malonyl hexoside (**10a**)	0.8 ± 0.3	BQL
***Flavonols***		
Quercetin pentoside 2 (**42**)	6.2 ± 0.2	7.1 ± 0.1
Quercetin glucuronide (**45**)	2.2 ± 0.3	0.9 ± 0.0
Quercetin pentoside 3 (**55**)	2.8 ± 0.0	2.4 ± 0.0

BQL: below quantification limit (for flavonols: 0.12 μg; for anthocyanins: 0.33 μg). Anthocyanins are expressed as equivalents of malvidin-3-*O*-glucoside and flavonols are expressed as equivalents of quercetin-3-*O*-glucoside/100 g fw.

**Table 5:** Anthocyanins, hydroxycinnamic acids and flavonols content of wild *Berberis* fruits from the Argentinean Patagonia (mg/100 g fw).

Compounds	*Berberis microphylla*					*Berberis darwinii*	
	Brazo Rincón	Aeropuerto	Villa La Angostura	Cuyín Manzano	Llanquín	Brazo Rincón	Villa La Angostura
***Anthocyanins***							
Delphinidin-3-glucoside (**1a**)	78.6 ± 0.9 ^a^	621.7 ± 9.1 ^b^	459.8 ± 5.8 ^c^	397.1 ± 0.9 ^d^	301.7 ± 1.6 ^e^	115.3 ± 2.7	163.3 ± 4.4
Delphinidin rutinoside (**2a**)	1.8 ± 0.3 ^a^	66.4 ± 3.5 ^b^	35.5 ± 1.7 ^c^	29.1 ± 1.0 ^d^	27.1 ± 0.3 ^d^	11.0 ± 0.1	10.9 ± 0.7
Cyanidin-3-glucoside (**3a**)	1.2 ± 0.0 ^a^	51.3 ± 3.4 ^b^	40.0 ± 1.7 ^c^	52.3 ± 1.1 ^b^	47.8 ± 2.6 ^b^	30.4 ± 0.0	68.2 ± 2.0
Cyanidin-3-rutinoside (**4a**)	BQL	BQL	BQL	BQL	BQL	BQL	2.3 ± 0.0
Petunidin hexoside (**5a**)	35.7 ± 1.0 ^a^	363.6 ± 6.0 ^b^	271.2 ± 1.4 ^c^	229.8 ± 0.8 ^d^	185.7 ± 9.7 ^e^	61.9 ± 0.1	83.7 ± 2.8
Petunidin rutinoside (**7a**)	1.0 ± 0.0 ^a^	78.5 ± 3.9 ^b^	37.2 ± 0.1 ^c^	30.3 ± 0.6 ^d^	33.5 ± 0.0 ^c,d^	7.3 ± 0.1	8.7 ± 0.9
Peonidin hexoside (**8a**)	BQL	11.2 ± 0.9 ^a^	6.4 ± 0.8 ^b^	15.3 ± 0.2 ^c^	16.5 ± 0.8 ^c^	1.2 ± 0.0	5.3 ± 0.7
Peonidin rutinoside (**9a**)	BQL	BQL	BQL	BQL	1.4 ± 0.1	BQL	BQL
Malvidin-3-glucoside (**11a**)	11.5 ± 0.4 ^a^	247.0 ± 3.6 ^b^	172.4 ± 0.2 ^c^	163.4 ± 0.3 ^d^	181.7 ± 4.8 ^e^	59.3 ± 0.8	66.8 ± 1.7
Malvidin rutinoside (**12a**)	BQL	54.5 ± 4.8 ^a^	21.9 ± 1.1 ^b^	17.4 ± 0.4 ^b^	35.6 ± 1.2 ^c^	4.3 ± 0.8	3.8 ± 0.7
***Hydroxycinnamic acids***							
Caffeoylglucaric acid 1 (**2**)	20.8 ± 0.6 ^a^	46.2 ± 1.6 ^b^	48.4 ± 0.3 ^c^	21.5 ± 0.4 ^a^	15.1 ± 0.3 ^d^	22.4 ± 0.4	96.3 ± 2.0
Caffeoylglucaric acid 2 (**5**)	9.2 ± 0.2 ^a^	23.3 ± 0.3 ^b^	27.4 ± 0.3 ^c^	46.3 ± 1.4 ^d^	22.3 ± 0.3 ^b^	59.2 ± 0.2	217.6 ± 1.4
Caffeoylquinic acid 3 (**13**)	163.7 ± 0.7 ^a^	74.1 ± 0.6 ^b^	163.4 ± 1.1 ^a^	35.2 ± 0.2 ^c^	31.6 ± 1.1 ^d^	100.0 ± 0.3	328.3 ± 2.2
Dicaffeoyl glucaric acid 2 (**14**)	28.2 ± 0.3 ^a^	20.0 ± 0.3 ^b^	56.3 ± 0.5 ^c^	25.2 ± 0.2 ^d^	17.6 ± 0.1 ^e^	7.1 ± 0.7	67.4 ± 0.1
Caffeoylquinic acid 3 (**16**)	3.2 ± 0.1 ^a^	4.9 ± 0.3 ^b^	6.2 ± 0.4 ^c^	6.8 ± 0.2 ^c^	4.2 ± 0.2 ^d^	7.6 ± 0.1	28.2 ± 0.2
Feruloylquinic acid (**18**)	6.3 ± 0.0 ^a^	17.3 ± 0.9 ^b^	16.2 ± 0.9 ^b^	23.3 ± 0.8 ^c^	12.6 ± 0.2 ^d^	6.8 ± 0.3	21.6 ± 0.5
Dicaffeoyl glucaric acid 3 (**24**)	2.1 ± 0.3 ^a^	1.8 ± 0.2 ^a^	2.8 ± 1.2 ^a^	5.4 ± 0.0 ^b^	7.1 ± 0.0 ^c^	27.2 ± 1.2	93.2 ± 0.1
Dicaffeoyl glucaric acid 6 (**36**)	2.8 ± 0.2 ^a^	3.3 ± 0.1 ^b^	6.4 ± 0.3 ^c^	5.2 ± 0.0 ^d^	9.5 ± 0.0 ^e^	24.1 ± 1.5	123.6 ± 3.4
Dicaffeoylquinic acid 7 (**51**)	16.5 ± 0.3 ^a^	11.6 ± 0.3 ^b^	20.3 ± 0.2 ^c^	9.6 ± 0.1 ^d^	4.5 ± 0.8 ^e^	10.4 ± 0.8	21.1 ± 0.5
***Flavonols***							
Quercetin hexoside (**43**)	10.5 ± 0.0 ^a^	41.5 ± 0.5 ^b^	24.6 ± 1.3 ^c^	31.2 ± 1.0 ^d^	26.2 ± 0.9 ^c^	29.8 ± 0.1	56.6 ± 2.5
Quercetin glucuronide (**45**)	ND	ND	2.8 ± 0.1	ND	ND	3.0 ± 0.3	13.6 ± 0.4
Quercetin rutinoside (**48**)	14.1 ± 0.0 ^a^	53.5 ± 2.7 ^b^	34.6 ± 0.5 ^c^	40.0 ± 0.9 ^d^	18.5 ± 1.2 ^e^	25.0 ± 0.7	57.3 ± 5.8
Quercetin acetylhexoside 1 (**52**)	12.2 ± 0.4 ^a^	BQL	20.7 ± 0.1 ^b^	7.1 ± 0.1 ^c^	5.4 ± 0.2 ^d^	25.1 ± 0.5	59.8 ± 1.7
Quercetin rhamnoside (**56**)	18.8 ± 0.6 ^a^	0.2 ± 0.3 ^b^	ND	4.2 ± 0.2 ^c^	1.5 ± 0.3 ^d^	28.0 ± 0.3	26.6 ± 1.1
Isorhamnetin rutinoside (**63**)	4.2 ± 0.1 ^a^	55.7 ± 2.6 ^b^	9.6 ± 0.9 ^a^	26.6 ± 3.1 ^c^	22.1 ± 2.2 ^c^	BQL	BQL
Isorhamnetin acetylhexoside 1 (**65**)	3.9 ± 0.0 ^a^	8.6 ± 0.5 ^b^	6.8 ± 0.0 ^c^	6.7 ± 0.2 ^c^	9.9 ± 0.2 ^d^	ND	ND
Isorhamnetin acetylhexoside 2 (**67**)	2.1 ± 0. 2^a^	6.8 ± 0.6 ^b^	6.5 ± 0.0 ^b^	6.5 ± 0.4 ^b^	3.2 ± 0.1 ^c^	ND	5.3 ± 0.4

ND: not detected; BQL: below quantification limit (for flavonols: 0.12 μg; for anthocyanins: 0.33 μg; for hydroxycinnamic acids: 0.07 μg). Different letters (^a–e^) in the same row show significant differences among each determination, according to Tukey’s test (*p* < 0.05). Anthocyanins are expressed as equivalents of malvidin-3-*O*-glucoside, hydroxycinnamic acids are expressed as equivalents of chlorogenic acid, and flavonols are expressed as equivalents of quercetin-3-*O*-glucoside/100 g fw.

**Table 6:** Pearson’s correlation coefficient for the content of polyphenols, antioxidant activity, and α-glucosidase inhibition.

Compound	DPPH	FRAP	TEAC	CUPRAC	ORAC	α-glucosidase
**Anthocyanins**						
**1a**	−0.866 **	0.884 **	0.851 **	0.832 **	0.796 **	−0.518 *
**2a**	−0.736 **	0.773 **	0.798 **	0.702 **	0.708 **	−0.311
**3a**	−0.630 *	0.675 **	0.991 **	0.664 **	0.992 **	−0.653 **
**5a**	−0.848 **	0.869 **	0.873 **	0.830 **	0.816 **	−0.512
**7a**	−0.674 **	0.709 **	0.784 **	0.633 *	0.682 **	−0.240
**8a**	0.675 *	−0.568	0.446	−0.717 **	0.607 *	0.104
**10a**	−0.678 **	0.706 **	0.962 **	0.687 **	0.900 **	−0.456
**11a**	0.078	0.002	0.774 **	−0.127	−0.150	0.765 **
**Hydroxycinnamic acids**						
**2**	−0.755 **	0.734 **	0.331	0.759 **	0.259	−0.212
**5**	−0.642 **	0.666 **	0.665 **	0.617 *	0.786 **	−0.957 **
**13**	0.074	−0.133	−0.664 **	−0.030	−0.682 **	0.436
**14**	−0.380	0.311	−0.091	0.489	−0.052	−0.285
**16**	−0.799**	0.825 **	0.649 **	0.842 **	0.755 **	−0.952 **
**18**	−0.795 **	0.837 **	0.790 **	0.765 **	0.870 **	−0.918 **
**24**	0.287	−0.236	0.385	−0.167	0.454	−0.242
**36**	0.176	−0.178	0.471	0.012	0.482	−0.150
**51**	−0.265	0.216	−0.439	0.298	−0.428	0.016
**Flavonols**						
**43**	−0.712 **	0.768 **	0.887 **	0.659 **	0.839 **	−0.476
**48**	−0.901 **	0.938 **	0.689 **	0.816 **	0.665 **	−0.564 *
**52**	−0.441	0.383	−0.186	0.498	−0.200	−0.045
**56**	0.614 *	−0.620 *	−0.987 **	−0.677 *	−0.944 **	0.498
**64**	−0.496	0.571 *	0.668 **	0.408	0.586 *	−0.167
**66**	−0.191	0.235	0.842 **	0.284	0.766 **	−0.138
**69**	−0.975 **	0.992 **	0.738	0.948 **	0.759 **	−0.779 **

* *p* < 0.05; ** *p* < 0.01, according to Pearson’s correlation coefficient.

## References

[1] Bvenura C., Sivakumar D. (2017). The role of wild fruits and vegetables in delivering a balanced and healthy diet. Food Res. Int..

[2] Jiménez-Garcia S.N., Guevara-Gonzalez R.G., Miranda-López R., Feregrino-Perez A.A., Torres-Pacheco I., Vazquez-Cruz M.A. (2013). Functional properties and quality characteristics of bioactive compounds in berries: Biochemistry, biotechnology, and genomics. Food Res. Int..

[3] Nile S.H., Park S.W. (2014). Edible berries: Bioactive components and their effect on human health. Nutrition.

[4] Schmeda-Hirschmann G., Jiménez-Aspee F., Theoduloz C., Ladio A. (2019). Patagonian berries as native food and medicine. J. Ethnopharmacol..

[5] Chamorro M.F., Ladio A.H., Molares S., Martínez J., Muñoz-Acevedo A., Rai M. (2018). Patagonian Berries: An ethnobotanical approach to exploration of their nutraceutical potential. Ethnobotany: Local Knowledge and Traditions.

[6] Speisky H., López-Alarcón C., Gómez M., Fuentes J., Sandoval-Acuña C. (2012). First web-based database on total phenolics and oxygen radical absorbance capacity (ORAC) of fruits produced and consumed within the south Andes region of South America. J. Agric. Food Chem..

[7] Arena M.E., Lencinas M.V., Radice S. (2018). Variability in floral traits and reproductive success among and within populations of *Berberis microphylla* G. Forst., an underutilized fruit species. Sci. Hortic..

[8] Abbasi A.M., Shah M.H., Li T., Fu X., Guo X., Liu R.H. (2015). Ethnomedicinal values, phenolic contents and antioxidant properties of wild culinary vegetables. J. Ethnopharmacol..

[9] Srivastava S., Srivastava M., Misra A., Pandey G., Rawat A. (2015). A review on biological and chemical diversity in *Berberis* (Berberidaceae). EXCLI J..

[10] Mokhber-Dezfuli N., Saeidnia S., Gohari A., Kurepaz-Mahmoodabadi M. (2014). Phytochemistry and pharmacology of berberis species. Pharmacogn. Rev..

[11] Gundogdu M. (2013). Determination of antioxidant capacities and biochemical compounds of *Berberis vulgaris* L. Fruits. Adv. Environ. Biol..

[12] Hassanpour H., Alizadeh S. (2016). Evaluation of phenolic compound, antioxidant activities and antioxidant enzymes of barberry genotypes in Iran. Sci. Hortic..

[13] Ersoy N., Kupe M., Sagbas H.I., Ercisli S. (2018). Physicochemical diversity among barberry (*Berberis vulgaris* L.) fruits from Eastern Anatolia. Not. Bot. Hort. Agrobot. Cluj-Napoca..

[14] Ruiz A., Hermosín-Guitérrez I., Mardones C., Vergara C., Herlitz E., Vega M., Dorau C., Winterhalter P., von Baer D. (2010). Polyphenols and antioxidant activity of calafate (*Berberis microphylla*) fruits and other native berries from southern Chile. J. Agric. Food Chem..

[15] Ramirez J.E., Zambrano R., Sepúlveda B., Kennelly E.J., Simirgiotis M.J. (2015). Anthocyanins and antioxidant capacities of six Chilean berries by HPLC-HR-ESI-ToF-MS. Food Chem..

[16] Ladio A.H., Lozada M. (2004). Patterns of use and knowledge of wild edible plants in distinct ecological environments: A case study of a Mapuche community from northwestern Patagonia. Biodivers. Conserv..

[17] Cheel J., Theoduloz C., Rodríguez J.A., Caligari P.D.S., Schmeda-Hirschmann G. (2007). Free radical scavenging activity and phenolic content in achenes and thalamus from *Fragaria chiloensis* ssp. *chiloensis*, *F. vesca* and *F*. x *ananassa* cv. Chandler. Food Chem..

[18] Jiménez-Aspee F., Thomas-Valdés S., Schulz A., Ladio A., Theoduloz C., Schmeda-Hirschmann G. (2016). Antioxidant activity and phenolic profiles of the wild currant *Ribes magellanicum* from Chilean and Argentinean Patagonia. Food Sci. Nutr..

[19] Jiménez-Aspee F., Theoduloz C., Ávila F., Thomas-Valdés S., Mardones C., von Baer D., Schmeda-Hirschmann G. (2016). The Chilean wild raspberry (*Rubus geoides* Sm.) increases intracellular GSH content and protects against H_2_O_2_ and methylglyoxal-induced damage in AGS cells. Food Chem..

[20] Ruiz A., Hermosín-Gutiérrez I., Vergara C., von Baer D., Zapata M., Hitschfeld A., Obando L., Mardones C. (2013). Anthocyanin profiles in south Patagonian wild berries by HPLC-DAD-ESI-MS/MS. Food Res. Int..

[21] Ruiz A., Mardones C., Vergara C., Hermosín-Gutiérrez I., von Baer D., Hinrichsen P., Rodríguez R., Arribillaga D., Domínguez E. (2013). Analysis of hydroxycinnamic acids derivatives in calafate (*Berberis microphylla* G. Forst) berries by liquid chromatography with photodiode array and mass spectrometry detection. J. Chromatogr. A.

[22] Ruiz A., Bustamante L., Vergara C., von Baer D., Hermosín-Gutiérrez I., Obando L., Mardones C. (2015). Hydroxycinnamic acids and flavonols in native edible berries of South Patagonia. Food Chem..

[23] Jiménez-Aspee F., Theoduloz C., Soriano M.D.P.C., Ugalde-Arbizu M., Alberto M.R., Zampini I.C., Isla M.I., Simirgiotis M.J., Schmeda-Hirschmann G. (2017). The native fruit *Geoffroea decorticans* from arid northern Chile: Phenolic composition, antioxidant activity and in vitro inhibition of pro-inflammatory and metabolic syndrome-associated enzymes. Molecules.

[24] Ruiz A., Mardones C., Vergara C., von Baer D., Gómez-Alonso S., Gómez M.V., Hermosín-Gutiérrez I. (2014). Isolation and structural elucidation of anthocyanidin 3,7-β-*O*-diglucosides and caffeoyl-glucaric acids from calafate berries. J. Agric. Food Chem..

[25] Lopes da Silva F., Escribano-Bailón M.T., Pérez Alonso J.J., Rivas-Gonzalo J.C., Santos-Buelga C. (2007). Anthocyanin pigments in strawberry. LWT-Food Sci. Technol..

[26] Simirgiotis M.J., Theoduloz C., Caligari P.D.S., Schmeda-Hirschmann G. (2009). Comparison of phenolic composition and antioxidant properties of two native Chilean and one domestic strawberry genotypes. Food Chem..

[27] Clifford M.N., Johnston K.L., Knight S., Kuhnert N. (2003). Hierarchical scheme for the LC-MS^n^ identification of chlorogenic acids. J. Agric. Food Chem..

[28] Cheel J., Theoduloz C., Rodríguez J.A., Saud G., Caligari P.D.S., Schmeda-Hirschmann G. (2005). *E*-cinnamic acid derivatives and phenolics from Chilean strawberry fruits, *Fragaria chiloensis* ssp. *chiloensis*. J. Agric. Food Chem..

[29] Schuster B., Herrmann K. (1985). Hydroxybenzoic and hydroxycinnamic acid derivatives in soft fruits. Phytochemistry.

[30] Jiménez-Aspee F., Theoduloz C., Gómez-Alonso S., Hermosín-Gutiérrez I., Reyes M., Schmeda-Hirschmann G. (2018). Polyphenolic profile and antioxidant activity of meristem and leaves from “chagual” (*Puya chilensis* Mol.), a salad from central Chile. Food Res. Int..

[31] Quatrin A., Pauletto R., Maurer L.H., Minuzzi N., Nichelle S.M., Carvalho J.F.C., Maróstica Junior M.R., Rodrigues E., Bochi V.C., Emanuelli T. (2019). Characterization and quantification of tannins, flavonols, anthocyains and matrix-bound polyphenols from jaboticaba fruit peel: A comparison between *Myrciaria trunciflora* and *M. jaboticaba*. J. Food Compos. Anal..

[32] Thomas-Valdés S., Theoduloz C., Jiménez-Aspee F., Schmeda-Hirschmann G. (2019). Effect of simulated gastrointestinal digestion on polyphenols and bioactivity of the native Chilean red strawberry (*Fragaria chiloensis* ssp. *chiloensis* f. patagonica). Food Res. Int..

[33] McDougall G.J., Kulkarni N.N., Stewart D. (2009). Berry polyphenols inhibit pancreatic lipase activity in vitro. Food Chem..

[34] Lin L.Z., Sun J., Chen P., Monagas M.J., Harnly J.M. (2014). UHPLC-PDA-ESI/HRMS^n^ profiling method to identify and quantify oligomeric proanthocyanidins in plant products. J. Agric. Food Chem..

[35] Chung S.W., Yu D.K., Lee H.J. (2016). Changes in anthocyanidin and anthocyanin pigments in highbush blueberry (*Vaccinium corymbosum* cv. Bluecrop) fruits during ripening. Hort. Environ. Biotechnol..

[36] Parra-Palma C., Fuentes E., Palomo I., Torres C.A., Moya-León M.A., Ramos P. (2018). Linking the platelet anti-aggregation effect of different strawberries species with antioxidants: Metabolomic and transcript profiling of polyphenols. BLACPMA.

[37] Xu C., Zhang Y., Zhu L., Huang Y., Lu J. (2011). Influence of growing season on phenolic compounds and antioxidant properties of grape berries from vines grown in subtropical climate. J. Agric. Food Chem..

[38] Arena M.E. (2016). Estudio de algunos fenómenos morfofisiológicos y cambios bioquímicos en *Berberis microphylla* G. Forst (sinónimo *B. buxifolia*) asociados a la formación y maduración de frutos en Tierra del Fuego y su relación con la producción de metabolitos útiles. Ph.D. Thesis.

[39] Williamson G. (2013). Possible effects of dietary polyphenols on sugar absorption and digestion. Mol. Nutr. Food Res..

[40] Törrönen R., Kolehmainen M., Sarkkinen E., Mykkänen H., Niskanen L. (2012). Postprandial glucose, insulin, and free fatty acid responses to sucrose consumed with blackcurrants and lingonberries in healthy women. Am. J. Clin. Nutr..

[41] Reyes-Farias M., Vasquez K., Fuentes F., Ovalle-Marin A., Parra-Ruiz C., Zamora O., Pino M.T., Quitral V., Jimenez P., Garcia L. (2016). Extracts of Chilean native fruits inhibit oxidative stress, inflammation and insulin-resistance linked to the pathogenic interaction between adipocytes and macrophages. J. Funct. Foods.

[42] Thomas-Valdés S., Theoduloz C., Jiménez-Aspee F., Burgos-Edwards A., Schmeda-Hirschmann G. (2018). Changes in polyphenol composition and bioactivity of the native Chilean white strawberry (*Fragaria chiloensis* spp. *chiloensis* f. chiloensis) after in vitro gastrointestinal digestion. Food Res. Int..

[43] Podsędek A., Majewska I., Redzynia M., Sosnowska D., Koziołkiewicz M. (2014). In vitro inhibitory effect on digestive enzymes and antioxidant potential of commonly consumed fruits. J. Agric. Food Chem..

[44] Favre G., González-Neves G., Piccardo D., Gómez-Alonso S., Pérez-Navarro J., Hermosín-Gutierrez I. (2018). New acylated flavonols identified in *Vitis vinifera* grapes and wines. Food Res. Int..

[45] ICH (2005). Validation of Analytical Procedures: Text and Methodology. https://www.ich.org/fileadmin/Public_Web_Site/ICH_Products/Guidelines/Quality/Q2_R1/Step4/Q2_R1__Guideline.pdf.

[46] Bondet V., Brand-Williams W., Berset C. (1997). Kinetics and mechanisms of antioxidant activity using the DPPH free radical method. LWT-Food Sci. Technol..

[47] Nenadis N., Wang L.F., Tsimidou M., Zhang H.Y. (2014). Estimation of scavenging activity of phenolic compounds using the ABTS^·+^ assay. J. Agric. Food Chem..

[48] Ou B., Hampsch-Woodill M., Prior R.L. (2001). Development and validation of an improved oxygen radical absorbance capacity assay using fluorescein as the fluorescent probe. J. Agric. Food Chem..

